# Archived HIV-1 Drug Resistance Mutations: Role of Proviral HIV-1 DNA Genotype for the Management of Virological Responder People Living with HIV

**DOI:** 10.3390/v16111697

**Published:** 2024-10-30

**Authors:** Roberta Campagna, Chiara Nonne, Guido Antonelli, Ombretta Turriziani

**Affiliations:** Department of Molecular Medicine, Sapienza University of Rome, 00185 Rome, Italy; chiara.nonne@uniroma1.it (C.N.); guido.antonelli@uniroma1.it (G.A.); ombretta.turriziani@uniroma1.it (O.T.)

**Keywords:** HIV-1, reservoir, DNA GRT, Sanger sequencing, NGS, APOBEC

## Abstract

Despite its effectiveness in controlling plasma viremia, antiretroviral therapy (ART) cannot target proviral DNA, which remains an obstacle to HIV-1 eradication. When treatment is interrupted, the reservoirs can act as a source of viral rebound, highlighting the value of proviral DNA as an additional source of information on an individual’s overall resistance burden. In cases where the viral load is too low for successful HIV-1 RNA genotyping, HIV-1 DNA can help identify resistance mutations in treated individuals. The absence of treatment history, the need to adjust ART despite undetectable viremia, or the presence of LLV further support the use of genotypic resistance tests (GRTs) on HIV-1 DNA. Conventionally, GRTs have been achieved through Sanger sequencing, but the advances in NGS are leading to an increase in its use, allowing the detection of minority variants present in less than 20% of the viral population. The clinical significance of these mutations remains under debate, with interpretations varying based on context. Additionally, proviral DNA is subject to APOBEC3-induced hypermutation, which can lead to defective, nonviable viral genomes, a factor that must be considered when performing GRTs on HIV-1 DNA.

## 1. Introduction

**Figure 1:**
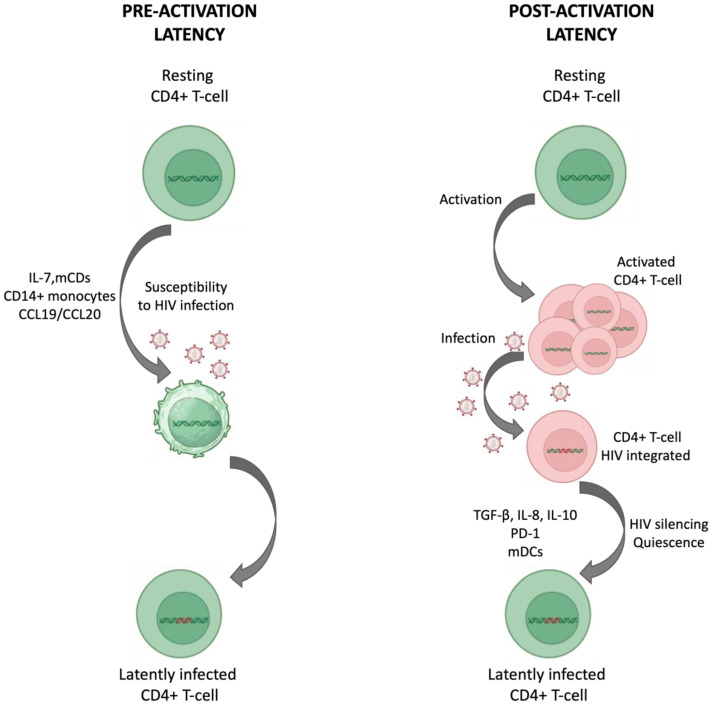
Main models proposed to explain the establishment of HIV-1 latently infected cells. Pre-activation latency: resting CD4+ T cells are refractory to HIV-1 infection, but under certain conditions such as the presence of IL-7, CCL-19, and CCL-20, the permissiveness of these cells to HIV-1 may increase, resulting in integrated proviruses. Post-activation latency: after activation, CD4+ T cells can become infected with HIV-1, but a subset of activated and infected CD4+ T cells can return to a resting state that allows integration and persistence of the viral genome. The presence of certain molecules can favor this transitional state. For instance, PD-1 dampens T-cell activation and the presence of myeloid dendritic cells (mDCs) can favor the transition to a post-activation latency state. mDCs: myeloid dendritic cells; PD-1: programmed cell death protein 1; TGF-β: transforming growth factor. Adapted from “The multifaceted nature of HIV latency” by Dufour et al. J ClinInvest. 2021; 131(11): e151380. doi: 10.1172/JCI151380 [4]. The above image was created with https://www.biorender.com/ (accessed on 28 July 2024).

For most people living with HIV (PLWH), antiretroviral therapy (ART) can suppress and maintain HIV-1 viral load (VL) below the detection of conventionally available tests leading to a reduction of morbidity, mortality, and HIV-1 transmission. However, despite this success, ART is not curative, as in the vast majority of PLWH, interruption of ART generally results in a rapid viremia rebound [1]. The origin of viral rebound is a pool of anatomically dispersed resting memory CD4+ T cells that harbor latent but inducible HIV-1 DNA. The pool of latently infected CD4+ T cells is established early in the course of the disease, during primary infection [2]. Memory CD4+ T cells are recognized as the major cellular reservoir for HIV-1 [3,4]. Some evidence suggests that a post-activation latency and a pre-activation latency are responsible for the establishment of the reservoir [4] (Figure 1). Post-activation latency is due to the ability of activated infected CD4+ T cells to revert to a resting state in which the integrated viral genome persists indefinitely. In the pre-activation latency model, HIV-1 directly infects resting CD4+ T-cells. Resting cells are usually refractory to productive infection, but under certain conditions, their susceptibility to the virus may increase. Over the last decade, the numerous research studies conducted to better understand the nature of viral reservoirs have led to a new view of HIV-1 persistence [5]. A much more dynamic and complex picture has thus emerged than previously believed.

Historically, the main cellular reservoir has always been associated with resting CD4+ T cells characterized by a long half-life; recent studies have shown that in PLWH undergoing treatment, proviral DNA is frequently found in CD4+ T cells that show a short half-life such as transitional and effector memory. Moreover, many individuals on ART express the activation marker HLA-DR, which is poorly compatible with a quiescent state [6]. These observations, therefore, suggest that the HIV-1 reservoir continues to proliferate also during therapy and that the longevity of the infected cell is not the only factor responsible for persistence. Currently, it is believed that the massive clonal expansion of cells harboring proviral DNA is responsible for the maintenance of infected cells during ART [7,8]. In PLWH starting ART in a chronic infection, viral reservoirs are generally larger and more genetically different than in individuals starting ART in an acute infection, consistent with the hypothesis that the reservoir is continuously formed during the untreated infection.

Several studies on PLWH in whom ART was successful but with a history of drug resistance provided evidence of the dynamic nature of the latent reservoir and demonstrated that any viral variant, including drug-resistant ones, can persist within reservoirs [9,10,11]. The finding that the proviral DNA may contain an archive of drug-resistant variants stimulated the interest of researchers who sought to understand whether these variants could pose a real threat to the success of therapy and whether the HIV-1 DNA could be a valid tool for assessing resistance in virologically suppressed individuals.

This review aims to provide an update on the main advances in the knowledge of archived drug-resistant viral variants, with a focus on tools available to detect drug-resistance mutations in proviral DNA and on their significance in the virological failure of treated individuals with controlled viremia.

## 2. HIV-1 Drug-Resistant Variants in Blood Compartments of ART-Treated PLWH

**Figure 2:**
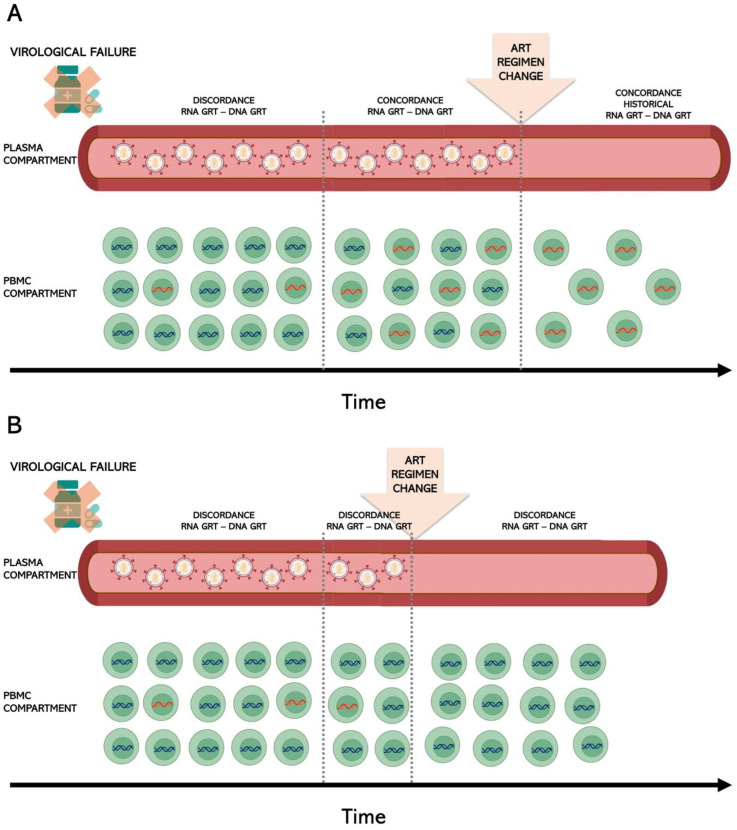
Concordance/discordance between RNA and DNA genotypic resistance test in PLWH with at least one virological failure. In the early stages of virological failure, the detection of emerging drug resistance mutations (DRMs) is more sensitive in viral RNA than in proviral DNA. This is because the drug-resistant virus has not yet had time to be archived in the reservoir. However, if the drug-resistant virus has been circulating for a sufficient period, allowing resistant viral variants to be stored in the reservoir, there may be good concordance between RNA and DNA genotyping (panel **A**). When the ART regimen is changed, the resistant viral variants may only be found in the reservoir. Conversely, if the drug-resistant virus has not circulated long enough, it will not have been archived in the reservoir (panel **B**). In the image, PBMCs with drug-resistant provirus are represented with orange DNA, while wild-type PBMCs are represented with blue DNA. The above image was created with https://www.biorender.com/ (accessed on 3 August 2024).

Several studies on ART-treated subjects have emphasized the value of proviral DNA as an additional source of information on the total burden of resistance in an individual [12,13,14]. The first evidence of the presence of drug resistance mutation in the proviral DNA dates back 20 years, when different authors analyzed the viral DNA from peripheral blood mononuclear cells (PBMCs) [15,16,17,18]. Most of these studies were conducted on samples derived from PLWH in ART failure with a history of drug resistance and showed that such drug-resistant variants can easily be found archived in PBMCs. Some authors observed a good concordance between PBMCs and plasma drug resistance profiles [15,19], but it was also observed that the PBMC compartment did not necessarily reflect the plasma compartment [16,17,20]. Different mutation dynamics in proviral DNA and plasma RNA are responsible for the discordance observed between these two districts in the different studies. A high concordance can be observed in individuals with a high level of viral replication and in whom the drug-resistant virus has circulated for a sufficient period to allow the resistant viral variants to be stored in the reservoir. The difference in drug resistance between RNA and DNA sequences in treatment-experienced individuals might be due to different factors. Resistance mutations emerge in plasma before they are detected in cells [21], but they can persist longer in the cellular compartment because there they are less sensitive to selective pressure (Figure 2).

Conversely, some authors reported that the detectability of archived DNA genotypic resistance may decrease over time [22] and others also suggested that cells harboring archived resistant provirus could be diluted by more recent uninfected cells and, therefore, be less readily detectable [23].

Furthermore, several studies have shown that standard genotyping of HIV-1 DNA is less informative than the entire history of RNA genotyping test results [historical plasma genotypic resistance tests (GRTs)] for documenting drug resistance mutations (DRMs) and guiding future therapeutic switches in virologically suppressed individuals [23,24]. In a multicenter randomized noninferiority trial, Delaugerre et al. conducted HIV-1 DNA resistance genotyping on 169 individuals before randomization and compared the findings to previous RNA genotyping results. The DNA analysis revealed significantly fewer resistance mutations in the reverse transcriptase (RT) and protease genes compared to the cumulative mutations detected through RNA testing [23].

In the perspective of a switch to the combination rilpivirine/emtricitabine/tenofovir disoproxil fumarate, Lambert-Niclot et al. evaluated whether proviral DNA was a potentially useful alternative to HIV-1 RNA for resistance genotyping in treated PLWH with at least 1 year of undetectable viremia. They studied individuals without virological failure (VF) and with at least one VF and compared viral DNA and historical RNA sequences of the RT gene. In PLWH, without prior VF, a good concordance was found between DNA and RNA genotype. Conversely, in individuals with at least one VF, the historical RNA genotype was more informative than the proviral DNA, suggesting that some resistance mutations had not been archived or that their levels had fallen below the detection threshold [25]. However, other authors have reported that, although resistance might be underestimated when detected in PBMCs, genotyping of proviral DNA can predict future virologic failure [26].

In the initial phase of virological failure, the detection of emerging DRMs is more sensitive in viral RNA than in proviral DNA; however, when the viral load is undetectable or shows levels that preclude the success of HIV-1 RNA genotyping, HIV-1 DNA may be useful to detect the resistance mutations.

Certain factors of the individual may be considered motivating factors for the use of GRTs on HIV-1 DNA, such as a lack of treatment history, the need for a change of ART when viremia is undetectable, the presence of low levels of viremia (LLV). Over the last decade, LLV, defined as a viral load between 50–1000 copies/ml, has been the subject of numerous studies. Although the clinical implication of LLV and its impact on clinical prognosis remains uncertain [27], the continuous process of HIV-1 evolution and the accumulation of DRMs during LLV is well recognized [28,29]. People with LLV have a higher risk of virological failure, developing DRMs, and immunological deterioration due to persistent low-level viral replication [30,31,32]. Several authors have attempted to assess the potential benefit of GRTs performed on PBMCs of drug-experienced PLWH with suppressed or low viremia. Zaccarelli et al. compared the resistance profile of proviral-DNA with historical plasma GRT [33]. They found that the detection of DRM in PBMCs from subjects with LLV was less frequent than in historical RNA genotypes, but analysis of proviral DNA showed the presence of at least one resistance mutation not detected in previous plasma GRTs. Furthermore, GRTs on PBMCs from individuals with LLV revealed the presence of mutations that conferred a certain degree of resistance to the therapeutic regimens administered [34]. Other researchers suggested that to optimize ART in PLWH with LLV, the antiretroviral resistance profile should be evaluated in combination with proviral DNA and RNA genotyping and a thorough history of previous treatment [35]. The utility of GRTs on HIV-1 DNA in individuals with LLV was also emphasized in a recent study in which the authors investigated the prevalence of LLV-associated DRMs by analyzing HIV-1 proviral DNA when HIV-1-RNA-based amplification failed [36]. These findings strongly suggest that PBMC genotyping may provide useful information to guide therapeutic choices in the case of low/undetectable HIV-1 RNA, especially when information on previous therapeutic regimens and/or resistance is lacking.

As above reported, in most of the studies, GRTs on proviral DNA underestimate resistance detection in PLWH under prolonged virological suppression; however, the introduction of next-generation sequencing (NGS) can increase sensitivity since this sequencing method can detect also minority variants that are not detected by the Sanger sequencing method.

A recent study on 20 heavily treated participants of the PRESTIGIO registry, experiencing virological failure, compared RNA and DNA GRTs at the time of failure performed by NGS with historical RNA GRT results obtained by Sanger sequencing. The concordance between RNA and DNA GRTs at failure was 66.3%. NGS confirmed historical resistance mutations, many of which were detected as low-frequency minority species only on DNA, highlighting the importance of testing PBMCs at failure to identify historical resistance that may no longer be present in plasma [37]. The same authors also found that in the case of virological suppression, NGS on proviral DNA can be useful not only in detecting resistance mutations previously found in historical plasma RNA but also in detecting new resistance never observed before [38]. In the study by Ellis et al., the authors reported the clinical outcomes related to the use of DNA GRTs to guide ART adjustment. They observed that proviral DNA GRTs provided additional information useful for simplifying switching ART in treatment-experienced individuals. Changes in ART guided by DNA GRTs did not lead to virological failure and likely contributed to improved long-term safety and quality of life [39].

In the framework of the TANGO study, archived pre-existing drug resistance mutations were assessed to investigate their impact on virologic response through 144 weeks. TANGO is a randomized open-label study in which subjects with virologic suppression on a standard 3-drug tenofovir alafenamide-based regimen (TBR) either switched to dolutegravir (DTG)/lamivudine (3TC) or continued their baseline ART. The TANGO study demonstrated that switching to DTG/3TC was non-inferior to continuing a regimen (TBR) in maintaining viral suppression in ART-experienced adults with HIV-1 through 144 weeks. In the study by Wang et al., it was observed that participants with major nucleoside reverse transcriptase inhibitors (NRTI), non-nucleoside reverse transcriptase inhibitors (NNRTI), protease inhibitors (PI), or integrase strand transfer inhibitors (INSTI) DRMs in proviral DNA had a similar high virologic response, including four individuals with archived M184V/I on DTG/3TC and three with archived M184V/I, as well as two with archived K65N/R on TBR [40]. Therefore, as other authors had observed [41,42], in this study, the presence of archived mutations did not affect the response to “recycled” drugs. These results seem to disagree with other studies reporting that the presence of DRMs in proviral DNA can predict virological failure. For instance, Armenia et al. observed that archived mutations together with other parameters, such as low nadir CD4 count and a short previous virological control, predicted virological rebound after ART switching in virologically suppressed individuals [26]. Cutrell’s team found similar findings [43]. These authors performed an analysis using pooled data from long-acting cabotegravir (CAB)+ rilpivirine (RPV) participants in FLAIR, ATLAS, and ATLAS-2M studies to investigate potential factors predictive of virological failure at week 48. They found that the presence of at least two of the following factors, proviral mutations of resistance to RPV, viral subtype A6/A1, and/or a BMI of at least 30 kg/m^2^, were associated with an increased risk of virological failure. Furthermore, in the ATLAS-2M study, it was observed that archived E138A mutation was present in five of the eight participants with confirmed virological failure [44]. Recently it has also been reported that the most common RPV-mutation in PBMCs from ART-naive and ART-suppressed PLWH was E138A. Overall, these observations suggest that the presence of E138A in proviral DNA might be one of the predictive factors for CAB/RPV failure. However, there is a need for further clinical studies on CAB/RPV to better define the role of archived E138A on virological outcome.

Furthermore, it is important to bear in mind that the impact of mutations present in proviral DNA on virological failure depends on several factors, such as the type of mutation, mutation abundance, components of the switch regimen, and the duration of virologic suppression. Mutations that are known to be responsible for reduced viral fitness may have greater difficulty re-emerging from the reservoir upon recycling of the drug that selected the mutation itself. The time of viral suppression seems to affect the persistence of resistant viral variants in the reservoirs. In fact, the proportion of drug-resistant viral variants stored in reservoirs has been shown to decrease over time in individuals with sustained virologic suppression [45]. Recently, Teyssou et al. investigated, in PLWH with fully suppressed viremia, the kinetics of M184V mutation reduction in proviral DNA and factors associated with M184V mutation clearance over time. They found that after 2.5 years, M184V was lost by 50% of PLWH. In addition, univariate analyses showed that a higher nadir CD4 count and lower zenith HIV-1 RNA viral load were correlated with faster mutation clearance [46]. A very interesting aspect of this study is that the presence of lamivudine/emtricitabine in the ART treatment regimen during the 5-year observation period was not associated with the persistence of M184V. Data from the PRESTIGIO study highlighted the importance of the frequency of the DRMs in proviral DNA. It was observed that only minority resistant variants with frequencies ranging from 5% to 20% seem to be relevant [38]. In fact, the number of these mutations was higher among people who later experienced virological rebound compared with those who maintained suppression. It follows from the above that several factors must be considered when assessing the clinical significance of archived DRMs, and it should not be forgotten that proviral DNA genotyping may include resistance mutations identified in defective (replication-incompetent) viruses as well as the presence of APOBEC mutations. These latter can further complicate the interpretation of the GRTs on PBMCs, especially when using NGS technology, as NGS can overestimate APOBEC hypermutation-induced variants depending on reporting thresholds.

## 3. The Potential Role of APOBEC Mutations in the Interpretation of Archived Drug Resistance Variant

Apolipoprotein B mRNA-editing enzyme catalytic polypeptide-like (APOBEC) 3G (A3G) is one of the first restriction factors discovered with anti-HIV activity [47]. Restriction factors are cell elements that inhibit viral replication at different stages of the viral replication process [48].

APOBEC proteins are a protein family that, in humans, consists of 11 primary members of enzymes that deaminate cytidines into uridines in DNA and/or RNA. Each APOBEC protein contains one or two copies of a cytidine deaminase domain [49]. APOBEC proteins expression is tissue- and cell-specific and can be stimulated by different triggers. Interferons are among the primary stimulators of APOBEC expression in cells such as natural killer cells, activated T cells, monocytes, and macrophages [50].

**Figure 3:**
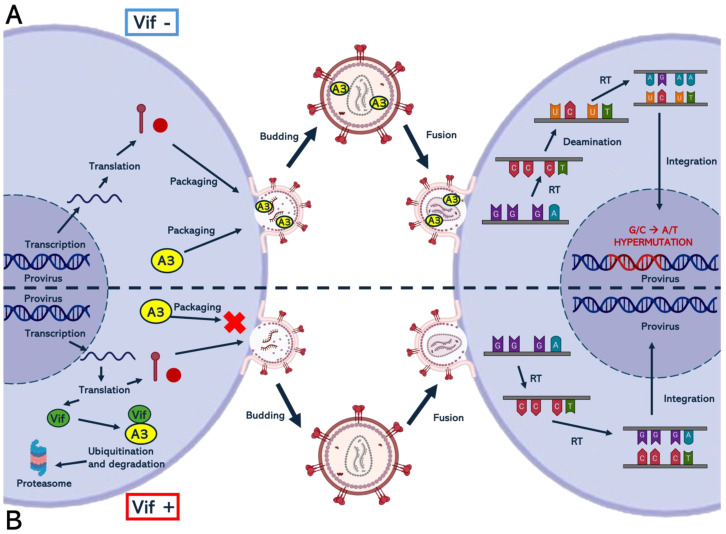
Mechanism of action of APOBEC3 (A3) against HIV-1 infection and Vif countermeasure. In the absence of Vif, (Vif −) A3 proteins are incorporated into the new virions by binding HIV-1 genomic RNA and the nucleocapsid protein. RNase H-dependent degradation of the HIV-1 RNA template during the reverse transcription phase exposes newly synthesized single-stranded DNA (ssDNA). A3 proteins detach from the genomic RNA, bind the nascent ssDNA, and deaminate cytidine into uridine (C-to-U) on the new ssDNA inducing high rates of G-to-A mutation in the newly synthesized plus strand of viral DNA (panel **A**). In the presence of Vif (Vif +), the formation of an E3 ubiquitin ligase complex is promoted, which targets A3 proteins for proteasomal degradation (panel **B**). A3 is represented in yellow; Vif is represented in green. The above image was created with https://www.biorender.com/ (accessed on 12 August 2024).

A3G is naturally expressed at significantly higher levels than other A3 family members and contributes to most of the APOBEC anti-HIV activity in T cells [49]. The antiviral activity of APOBEC3 (A3) proteins in blocking viral replication was confirmed in various studies that examined the effects of the lack of viral infectivity factor (Vif) expression in HIV-1 [51,52,53,54]. In the absence of Vif, A3 proteins bind HIV-1 genomic RNA and the nucleocapsid protein, allowing them to be incorporated into the new virions [47,55]. Several studies have shown that A3 proteins preferentially bind HIV-1 genomic RNA over cellular RNA [56]. During the reverse transcription phase, RNase H-dependent degradation of the HIV-1 RNA template exposes newly synthesized single-stranded DNA (ssDNA) [57]. A3 proteins detach from the genomic RNA and bind the nascent ssDNA, where they exert their deoxycytidine deaminase activity, converting deoxycytidine into deoxyuridine (C-to-U) on the new ssDNA [58]. This activity of A3 is termed hypermutation because it induces high rates of G-to-A mutation in the newly synthesized plus strand of viral DNA, resulting in defective nonviable integrated hypermutated viral genomes [59] (Figure 3).

The hypermutation activity of A3 proteins is highly dependent on the nucleotide context. In particular, A3G causes GG to be mutated to GA, and APOBEC3F(A3F) causes GA to be mutated to AA [60]. The resulting genomes often contain stop codons due to A3G editing of tryptophan and resulting from A3F editing of aspartic acid or glutamic acid [61].

The level of A3 restriction is proportional to enzyme expression and packaging into virions. This conclusion is supported by the observation that PLWH with higher A3G expression have lower levels of circulating HIV-1 RNA and disease progression [62,63,64,65].

A3 proteins also inhibit HIV-1 replication through deamination-independent mechanisms in which it is hypothesized there is a direct binding of A3 proteins to viral genomic RNA that delays primer extension and sterically blocks the progression of reverse transcriptase. The result is reduced viral cDNA synthesis as well as defects in plus-strand DNA transfer and integration [66,67,68,69,70,71,72,73,74].

HIV-1 encodes the Vif protein to counteract the function of A3 proteins, preventing hypermutation and enabling efficient virion replication. Vif promotes the formation of an E3 ubiquitin ligase complex, which targets A3 proteins for proteasomal degradation [55]. This depletes the pool of A3 proteins available for incorporation into the assembling viral particle and thereby, minimizes their ability to restrict HIV-1 replication [55,75,76,77]. A second mechanism for Vif-mediated antagonism occurs through restricted translation of A3G mRNA. Vif inhibits the translation of A3G mRNA to further decrease its intracellular level in HIV-infected cells [78,79,80,81].

Some evidence indicates that A3 proteins neutralization by Vif is not always absolute [82,83,84]. In some cases, Vif loses its ability to effectively counteract A3 proteins, leading to an increase in G-to-A mutations in the viral genome [23].

APOBEC3-induced mutagenesis in HIV-1 virions can vary significantly: while lethal mutagenesis is negatively selected in vivo, moderate mutagenesis can induce sublethal changes that increase viral heterogeneity contributing to the development of drug resistance [82,85,86,87,88]. For instance, Fourati and colleagues in 2010 identified four polymorphic positions in the Vif protein (K22, Y30, L153, K157) associated with antiretroviral treatment failure, with a focus on the K22H mutation, which is significantly more frequent in individuals with ART failure. This mutation, located in a known functional domain of Vif, impairs the ability of Vif to degrade A3 proteins, thus promoting the selection of G-to-A resistance mutations [83].

Therefore, it is believed that sublethal editing of A3 proteins, while contributing only modestly to viral genetic diversity, may induce mutational fitness effects that facilitate the evolution of drug resistance [82,89,90].

In a European case-control study, Cozzi-Lepri et al. demonstrated that pre-existing minority drug-resistant variants (DRMVs) associated with resistance to reverse transcriptase inhibitors double the risk of virological failure in ART-naive people initiating regimens containing efavirenz or nevirapine. Notably, the six most prevalent mutations identified in the study were nucleotide substitutions from G-to-A, resulting in amino acid changes from valine to isoleucine (V90I, V106I, and V108I), methionine to isoleucine (M184I and M230I), or glutamate to lysine (E138K) [91]. In the aforementioned study, RNA sequencing was performed from plasma samples, but it should be noted that APOBEC3-induced mutagenesis is particularly prevalent in proviral DNA sequences [92,93]. Furthermore, several whole-genome virus sequencing studies have revealed that more than 90–95% of integrated HIV-1 proviruses are defective and exhibit extensive APOBEC3-mediated hypermutations [94,95,96,97]. These defective proviruses, characterized by premature stop codons and mutations in highly conserved regions, are completely or partially transcriptionally silent and are unable to sustain viral replication [94,96,98].

In PLWH who receive ART and are virologically suppressed, a large proportion of HIV-1 proviruses are defective [99]. DRMs resulting from G-to-A hypermutation tend to be present in nonfunctional viruses that also contain stop codons and other disabling mutations [13].

Mazouz et al. assessed the proportion of APOBEC3-induced defective proviruses in 104 virologically suppressed PLWH, evaluating 8 weeks before (W8) and 48 weeks after (W48) a switch from triple therapy to dolutegravir + lamivudine.

They observed that, after one year of treatment, there was no significant change in the proportion of individuals with APOBEC3-defective proviruses. However, at least one APOBEC3-related drug resistance mutations (APO-DRMs) was observed in proviruses of 27% and 38% of participants at W8 and W48, respectively, with a significant increase in the ratio of APO-DRMs/number of potential APO-DRMs sites at week 48 compared to 8 weeks before therapy initiation. Thus, while the overall proportion of defective proviruses did not change, the presence of drug resistance-related mutations increased, indicating an evolving proviral reservoir under dual therapy. Mazouz and colleagues’ findings emphasize the need for longer-term studies to assess the potential risks associated with the presence of drug-resistance mutations [100].

Interestingly, an 11-year retrospective study was conducted by Armenia et al. involving 1126 virologically suppressed HIV-1 drug-experienced individuals from 2010 to 2021. They observed that APOBEC editing was present in 21.8% of the participants, with 20.3% with APOBEC-mediated mutations (APO-M) and 9.3% with stop codons. Additionally, 17% of individuals carried at least one APO-DRM. However, it is crucial to highlight that several APO-DRMs were not consistently linked to APOBEC editing. The authors observed that, out of the 18 APO-DRMs cataloged in the Stanford database, 8 were correlated with the presence of stop codons. This finding suggests that considering all these mutations as drug resistance-related could potentially lead to an overestimation of resistance. Conversely, other APO-DRMs, detected with considerable prevalence, were not associated with either APO-M or stop codons. As a result, excluding these mutations from consideration could result in an underestimation of resistance [101].

In addition, over the 11-year study period, a significant increase in the prevalence of APO-M was noted. In contrast, the prevalence of stop codons and APO-DRMs remained relatively unchanged over time, suggesting a complex and multifaceted role for APOBEC editing in the viral evolution observed in these individuals [101].

Furthermore, when evaluating HIV-1 DNA sequencing methods, it is important to consider the differences between NGS and Sanger sequencing. For instance, Mazouz’s work utilized NGS, while Armenia’s study employed Sanger sequencing. In several studies, it has been suggested to set a threshold of 5% to balance a good sensitivity for detecting low-frequency DRMs while mitigating technical artifacts. If the threshold is set too low, it may result in detecting numerous atypical mutations that could be attributed to technical errors rather than true low-frequency variants. Therefore, selecting an appropriate detection threshold is essential for ensuring both accuracy and reliability in the identification of drug resistance without compromising sequence accuracy [38,102,103,104].

Ultimately, it should be mentioned that various approaches are used to identify hypermutated sequences associated with A3 protein activity. In the aforementioned Mazouz et al. work, bioinformatic algorithms were developed to identify APOBEC3-defective sequences and APO-DRMs. All hypermutated sequences and those containing at least one stop codon were considered as [100]. In contrast, in the Armenia et al. work, resistance interpretation and estimation of APOBEC editing in HIV-1 DNA were made using the Stanford algorithm (HIVdb version 9.1, https://hivdb.stanford.edu/). A sequence was considered affected by APOBEC editing when at least one APO-M or a stop codon was detected [101].

One tool frequently used in various studies is the Hypermut Program (Los Alamos). Hypermut assesses the extent of G-to-A substitutions by comparing them to a reference sequence using two methods: G-to-A burden, which compares the number of G-to-A substitutions to the number of G nucleotides in the reference sequence; and G-to-A preference, which compares the number of G-to-A substitutions to the total number of substitutions. Hypermut 2.0 enables also the dinucleotide context to be considered: GG-to-AG for A3G and AG-to-AA per A3F to measure substitutions G-to-A enzyme specific [61]. A challenge in using various approaches to identify hypermutated sequences associated with A3 protein activity is that they may either underestimate or overestimate the modifications introduced by A3. Some algorithms may fail to recognize all the modifications made by A3 and the heterogeneity of target sequences or methods that only consider specific mutations might lead to the exclusion of changes that could be indirect consequences of A3 activity. Additionally, certain approaches may attribute mutations to A3 activity even when they could be caused by other factors, such as DNA polymerase errors or external mutagens. When algorithms cannot clearly differentiate between A3-induced mutations and random ones, there is a risk of misinterpreting data and overestimating A3 activity impact [49].

Regardless of the approach used, DNA proviral sequences can be considered hypermutated if they show a global excess of G-to-A changes in the appropriate dinucleotide context [61] or if they contain 2, 3 or more typical APOBEC mutations in a single sequence [105]. Any DRM that occurs in a dinucleotide context that can be traced to APOBEC activity should be interpreted with caution.

However, excluding all DRMs resulting from GG-to-AG or GA-to-AA changes could reduce sensitivity in detecting drug resistance. Indeed, in the Stanford HIV Drug Resistance Database, there are described at least 18 DRMs that occur in these dinucleotide contexts [13,106,107]. These DRMs of interest associated with APOBEC3 activity include: D30N, M46I, G48S and G73S in protease; D67N, E138K, M184I, G190ES and M230I in reverse transcriptase; and G118R, E138K, G140RS, G163KR, D232N and R263K in integrase [107]. Among the most common DRMs associated with APOBEC3 activity are NRTI M184I, resistance mutation to PI M46I, resistance mutations to NNRTI E138K and G190S, and resistance mutations to INSTI G118R, G140S, and R263K [37].

Therefore, proviral DNA sequences that exhibit a high degree of hypermutation should be excluded from GRT analysis, as they are likely associated with defective viruses. However, among the remaining sequences, the DRMs resulting from APOBEC3 activity should still be considered. This is crucial to avoid underestimating the presence of resistance mutations in the proviral DNA sequences.

## 4. Sanger Versus Next Generation Sequencing for the Detection of Archived Drug-Resistance Mutations

The conventional tool used to detect drug resistance mutations has been for many years Sanger sequencing. This method generates a consensus sequence, reflecting the most frequently identified bases at each nucleotide position [108]. Currently, this method is characterized by high automation that, alongside its reproducibility and clinical validation, has facilitated its widespread application in GRTs for both RNA and DNA [109]. The standardization of Sanger sequencing over the years played a crucial role in delivering reliable, robust, and consistent results, which are essential for the proper clinical management of PLWH [110]. As previously stated, Sanger sequencing has been and still represents the most used tool for the identification of DRMs in both HIV-1 RNA and HIV-1 DNA samples. This method enables the detection of those viral variants represented in at least 20% of the total viral population within a sample [12,111]. As a result, the consensus sequence derived from Sanger sequencing will reflect only the predominant viral variants present in a sample. Hence, despite being reproducible and validated for clinical use, one of the main limitations of Sanger sequencing is that it does not show the minority variants represented in less than 20% of the viral population [112,113,114].

Although Sanger sequencing is still important for performing GRTs, advances in new technologies, particularly NGS, are leading to an increasing use of the latter. Unlike the chain terminating method, NGS is a massively parallel sequencing technology that allows the processing of millions of sequences in parallel [111,115,116]. Among the advantages offered by next-generation sequencing, is the possibility of multiplexing, which enables the simultaneous analysis of multiple samples, and reduces the overall costs per run when the number of samples is high [110,117]. In addition, compared to Sanger sequencing, NGS enables the revelation of low-level DRMs, thus allowing the detection of minority variants; specifically, it can detect viral variants with a frequency of about 2% [102,118,119,120,121].

In a work from 2018, Arias et al. analyzed the concordance between Sanger sequencing and NGS results in identifying drug resistance mutations in both plasma and CD4+ T cells. The authors found a strong correlation between the results obtained using the two methods when using a 20% threshold. However, when the threshold was lowered to 5%, NGS allowed the detection of major and minor DRMs [122]. Different authors reported that the detection of low-abundance variants could significantly affect clinical outcomes, facilitating the clinical management of PLWH and enhancing the treatment effectiveness [123,124]. Inzaule et al. compared the resistance tests of a group of PLWH who went into virological failure with a control group, finding a higher percentage of drug resistance mutations in the former group. By lowering the detection threshold from 20% to 5% and even to 1%, they were able to identify an increasing number of DRMs in those who later experienced virological failure, proving that NGS can enhance the sensitivity of resistance testing, improving the ability to identify subjects at higher risk of virological failure [125].

While this method permits an extremely high sequencing depth, for clinical applications a threshold of 5% or 10% is generally preferred, as lower values carry a higher risk of encountering bias and detecting artifacts [13,103,104]. One of the disadvantages of adopting NGS for GRTs is the initial cost of setting logistics and infrastructures, although subsequently, a higher throughput is achieved at a lower cost per test [12,126]. Among the various critical issues to be considered in the application of NGS, the large amount of data produced that may require bioinformatics expertise for interpretation should not be forgotten. Currently, several products have received regulatory approval for use as in vitro diagnostics (IVD) through NGS, such as DeepChek-HIV (ABL, Luxemburg), Sentosa SQ HIV (Vela Diagnostics, Singapore), and HIV-1 Solution v2 (Arrow Diagnostic, Genova, Italy). However, due to the significant costs involved, many laboratories opt to develop their own in-house tests, a decision made easier by the availability of free pipelines that enable the analysis of sequencing results. Standardizing these methods is essential to ensure consistent clinical applications [127].

Furthermore, as previously mentioned, NGS enhances the detection of DRMs compared to Sanger sequencing by allowing the identification of minority variants. However, the uneven distribution of the provirus within the cell compartment can impact NGS performance [128]. Indeed, the number of cells containing proviral HIV-1 DNA decreases during the first years of ART, remaining stable during prolonged virological suppression [129]. The success of the test depends, among other factors, on the levels of proviral DNA in PBMCs. The quantification of HIV-1 DNA levels as well as the PBMC count are infrequently assessed before performing GRTs [13]. The easiest approach for HIV-1 DNA quantification is through Real-time polymerase chain reaction (RT-PCR), which measures the total HIV-1 DNA but cannot differentiate between integrated and unintegrated forms [130]. Quantifying HIV-1 DNA before performing NGS on proviral DNA could avoid sequencing failure due to low intracellular viral DNA; however, to date, no HIV-1 DNA threshold has been recommended for successful NGS. Moreover, although much progress has been made in the quantification of HIV-1 DNA [131], this test is not currently included in the standard diagnostic routines.

The use of NGS as a tool for both proviral DNA and HIV-1 RNA GRTs is rapidly increasing; however, there are some aspects that need to be addressed to ensure a consistent clinical application. This technology requires complex laboratory and analytic methods that are not yet well standardized between laboratories, so it is essential to plan multi-center studies in order to standardize the different methods. Moreover, it would be desirable to have some basic criteria to best interpret the presence of the minority variants. To date, the clinical relevance of these mutations is still being debated and requires varied interpretations based on the specific context (e.g., naive people, virological failure, virologic suppression) [132,133]. It has been suggested that the detection threshold could also be adjusted according to the drug class or even the specific mutation identified, to avoid unnecessary changes in the treatment regimen [125,127].

Despite the progress made in the development and the accuracy of methods for GRTs, in resource-limited settings, these technologies may still be not widely affordable. In low- and middle-income countries (LMICs), the high cost of GTRs, the lack of adequate structures and trained personnel, along with the difficulty in specimen collection and storage, represent limitations for DRM monitoring and optimal management of PLWH. To address these challenges, the use of dried blood spots (DBSs) is one potential solution [134]. Additionally, employing bench-top NGS platforms for large-scale surveillance studies in LMICs could help lower costs associated with this technology while ensuring effective monitoring of DRMs in PLWH [117].

Finally, for the most complex clinical cases, in addition to GRTs, it is possible to perform also phenotypic resistance tests. This type of assay allows virus susceptibility to varying concentrations of antiretroviral drugs to be measured [135,136]. However, as this is costly and requires a long turnaround time, it is mostly used for drug development and drug-resistance studies [137].

## 5. Conclusions

The use of genotypic resistance tests for proviral DNA is becoming increasingly common. Although numerous studies have evaluated the presence of DRMs in PBMCS, the clinical significance of archived mutations remains to be defined. Some studies have shown that archived DRMs can predict virological failure, but there are aspects that need further investigation to define how to accurately interpret these mutations. The introduction of advanced technologies such as NGS, while allowing greater sensitivity in identifying archived DRMs, may complicate their interpretation, especially when resistant variants are detected as minority variants and when induced APOBEC mutations are identified.

The historical plasma genotype, which is more informative than GRTs on proviral DNA, certainly helps the clinician in choosing a therapeutic switch, but there are scenarios in which GRTs on proviral DNA, despite their limitations, prove to be a valuable tool. Certainly, in subjects with low or undetectable viremia and with previous unknown therapy and/or virological failures, who need a switch or a therapeutic simplification, DNA genotyping represents the only way to know whether resistant variants are present. However, it is important to emphasize that even when historical HIV-1 RNA genotyping is available, knowledge of archived resistance could provide a more complete picture of drug resistance and thus, assist the clinician in the management of PLWH.
